# Non-invasive hemodynamic diagnosis based on non-linear pulse wave theory applied to four limbs

**DOI:** 10.3389/fbioe.2023.1081447

**Published:** 2023-03-09

**Authors:** Xiaorui Song, Yi Liu, Sirui Wang, Honghui Zhang, Aike Qiao, Xuezheng Wang

**Affiliations:** ^1^ School of Radiology, Shandong First Medical University and Shandong Academy of Medical Sciences, Tai’an, China; ^2^ Department of Ultrasound, Taian Maternity and Child Health Care Hospital, Tai’an, China; ^3^ Graduate School of Engineering, Chiba University, Chiba, Japan; ^4^ College of Engineering, Inner Mongolia Minzu University, Tongliao, China; ^5^ Faculty of Environment and Life, Beijing University of Technology, Beijing, China; ^6^ Department of Medical Image, The Second Affiliated Hospital of Shandong First Medical University, Tai’an, China

**Keywords:** blood pressure, pulse wave, four limbs, non-linear pulse wave theory, non-invasive, hemodynamic diagnosis, cardiovascular function

## Abstract

**Introduction:** Hemodynamic diagnosis indexes (HDIs) can comprehensively evaluate the health status of the cardiovascular system (CVS), particularly for people older than 50 years and prone to cardiovascular disease (CVDs). However, the accuracy of non-invasive detection remains unsatisfactory. We propose a non-invasive HDIs model based on the non-linear pulse wave theory (NonPWT) applied to four limbs.

**Methods:** This algorithm establishes mathematical models, including pulse wave velocity and pressure information of the brachial and ankle arteries, pressure gradient, and blood flow. Blood flow is key to calculating HDIs. Herein, we derive blood flow equation for different times of the cardiac cycle considering the four different distributions of blood pressure and pulse wave of four limbs, then obtain the average blood flow in a cardiac cycle, and finally calculate the HDIs.

**Results:** The results of the blood flow calculations reveal that the average blood flow in the upper extremity arteries is 10.78 ml/s (clinically: 2.5–12.67 ml/s), and the blood flow in the lower extremity arteries is higher than that in the upper extremity. To verify model accuracy, the consistency between the clinical and calculated values is verified with no statistically significant differences (*p* < 0.05). Model IV or higher-order fitting is the closest. To verify the model generalizability, considering the risk factors of cardiovascular diseases, the HDIs are recalculated using model IV, and thus, consistency is verified (*p* < 0.05 and Bland-Altman plot).

**Conclusion:** We conclude our proposed algorithmic model based on NonPWT can facilitate the non-invasive hemodynamic diagnosis with simpler operational procedures and reduced medical costs.

## 1 Introduction

Cardiovascular diseases (CVDs) have been the cause of numerous deaths and a major public health concern worldwide (Moran et al., 2014; McAloon et al., 2016). However, the early clinical symptoms of CVDs in the cardiovascular system (CVS) are not apparent, and medical measures can only be implemented when symptoms or complications are detected. Therefore, this presents a large gap in treating CVDs. Studies have shown that cardiovascular functions are characterized by pulse waves changes in hemodynamic diagnosis indexes (HDIs) are reflected by changes in pulse waveforms, which can be used for the early detection of CVDs symptoms (Chow et al., 2008; O’Rourke, 2009; Celermajer et al., 2012; SONG and QIAO, 2015; Wang et al., 2022). HDIs can be used to comprehensively evaluate the health status of a patient’s CVS, particularly for patients older than 50 years and prone to CVDs, more accurately and directly in terms of total peripheral resistance (TPR), stroke volume (SV), arterial compliance (AC), etc., (Thenappan et al., 2015; Suzuki et al., 2019; Trammel and Sapra, 2020). However, the direct detection of HDIs requires specialized and expensive equipment along with the guidance of clinical technicians and can be invasive (Meyers et al., 2008; Tachibana et al., 2016), which hinders their clinical application.

Blood pressure and pulse wave have been used as the basis for diagnosing and treating CVDs in clinical practice owing to their convenience and rapid, highly reliable detection (Wilkinson et al., 2002; Santana et al., 2012). However, the CVS is complex, and the pulse wave information of a single limb cannot fully describe the health of a patient’s CVS. Studies (O’Shea and Murphy, 2000; Clark et al., 2012b; Mancia et al., 2013; Sughimoto et al., 2014; Singh et al., 2015; Wu et al., 2020) have further confirmed that the blood pressure and pulse wave of four limbs can be used to comprehensively evaluate the CVS and diagnose CVDs, thus providing more clinical signs of disease and aid doctors in clinical decision-making (van der Hoeven et al., 2013; Singh et al., 2015). The European Society of hypertension and society of Cardiology reported that the systolic blood pressure difference between two arms more than 10 or 15 mmHg can cause peripheral vascular diseases (Mancia et al., 2013; Weber et al., 2014). The International Institute of Clinical Health (Clark et al., 2012b) demonstrated that a blood pressure difference of over 20 mmHg between two arms is dangerous and typically related to potential CVDs for hypertension. Clark et al. (2012a) reported that patients with this difference require further vascular evaluation, thereby suggesting that this signal is a useful indicator for vascular diseases and risk of death. Chen, Su, Clark, and others (Clark et al., 2007; 2012b; 2012a; Chen et al., 2012; Sheng et al., 2013; Cao et al., 2014; Su et al., 2014) have reported that the upper limb diastolic pressure difference and lower limb diastolic pressure difference of more than 10 mmHg or 15 mmHg correspond to statistical differences in the statistical analysis of peripheral vascular disease, CVDs mortality, and all-cause mortality. Therefore, the signal data of blood pressure and pulse wave of four limbs must be used to improve the accuracy of the HDIs calculation model.

The non-invasive detection method and application of cardiovascular function based on pulse wave theory, from theoretical description to modeling analysis and from linearization to non-linear theory, have been developed (Li et al., 1981). Studies have found that the oscillometric method and arterial elastic lumen theory used to calculate pulse wave conduction velocity can be used to evaluate cardiovascular health status (Boutouyrie, 2008; Solà et al., 2011). A goat experiment was conducted to compare oscillometric non-invasive and invasive blood pressure measurements. The results revealed that the accuracy of oscillometric blood pressure measurement technology was still insufficient; it was a method suitable for groups rather than individuals. Previous studies developed a new model based on the non-linear pulse wave theory (NonPWT), constructed motion and constitutive equations of the blood vessel wall and peripheral tissues, and introduced animal experiments to estimate the non-linear coefficients of the pressure gradient and pulse wave propagation velocity (Hashizume, 1988; Wu and Lee, 1989; Ma et al., 1992; Dimitrova, 2015; Chen et al., 2017; Bi et al., 2021). This model preserves the waveform characteristics measured for blood vessel walls and peripheral tissues and improves estimation accuracy. To apply NonPWT for HDIs calculations, the blood pressure and pulse waveform data of four limbs are required. These data must be selected to accurately characterize the complexity of cardiovascular features. Simultaneously, NonPWT that can preserve the non-linear characteristics of the pulse wave must also be proposed.

In this study, a hemodynamic diagnosis model for the CVS was developed. Additionally, mathematical models, including the pulse wave velocity and pressure information of the brachial and ankle arteries, pressure gradient, and blood flow based on NonPWT, were established. Corresponding to blood flow, a mathematical model with signal data of blood pressure and pulse waveform of the four limbs was proposed. The consistency between the clinical and calculated values was determined to evaluate the performance of the mathematical models. Evidently, compared with the previous hemodynamic diagnosis method, the proposed hemodynamic diagnosis method is quicker and more effective in calculating HDIs (SV, AC, and TPR), which reflect the blood-pumping function of the heart and the elasticity of blood vessels. Further, its calculations are consistent with clinical measurements, and it can be used to non-invasively evaluate cardiovascular function. Unlike the traditional pulse wave theory used to calculate HDIs our method extracts the pulse waveform data of four limbs, and considers the risk factors of cardiovascular diseases; Hence, it has greater accuracy and can be used to calculate more HDIs more simply.

## 2 Methods

### 2.1 Data collection

**FIGURE 1:**
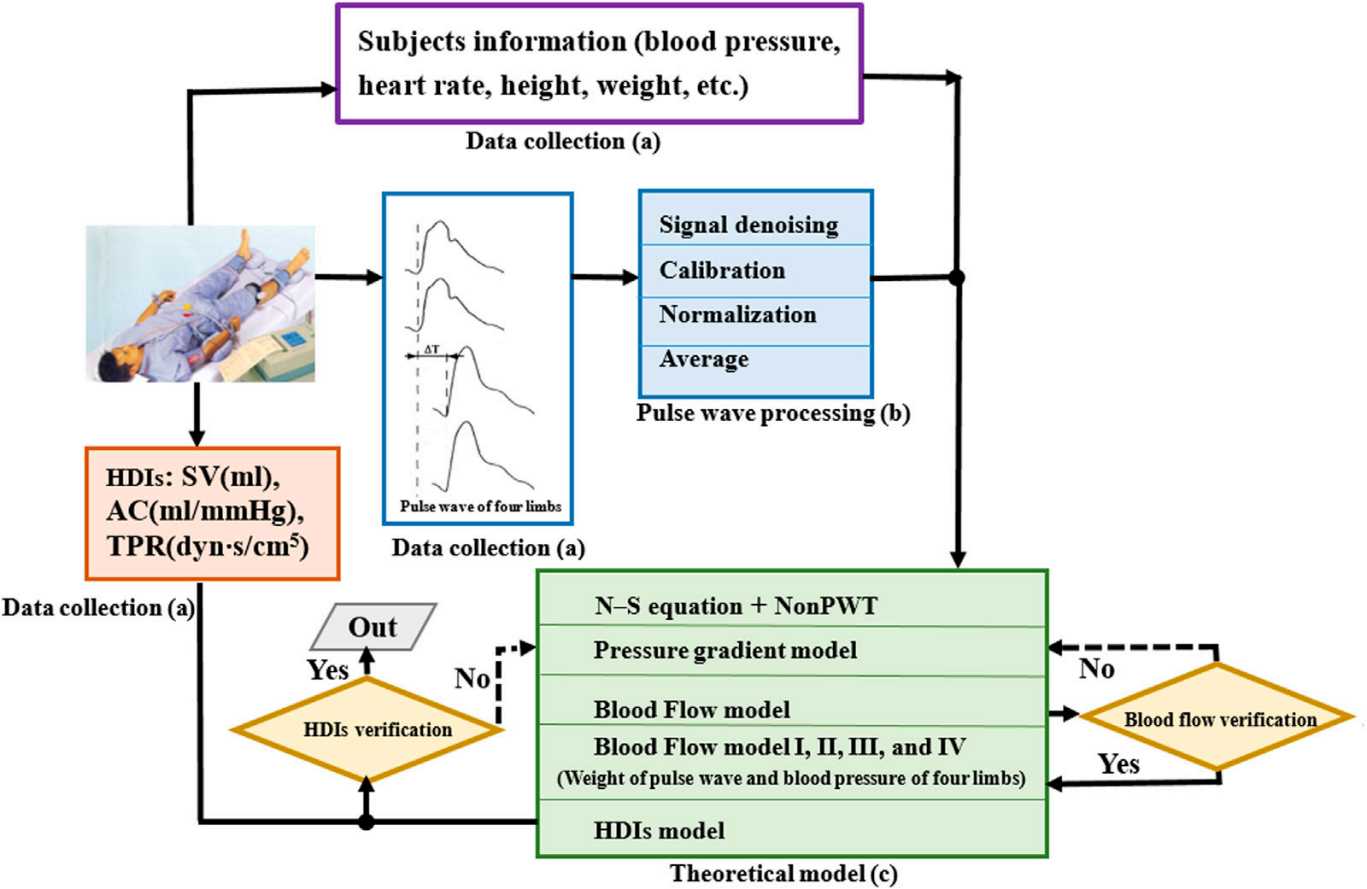
Construction process of hemodynamic diagnosis indexes model. **(A)** Data collection, **(B)** pulse wave processing, and **(C)** theoretical model. HDIs, hemodynamic diagnosis indexes; SV, stroke volume; TPR, total peripheral resistance; AC, arterial compliance.

The study, its experimental protocols, and relevant details were approved by the Institutional Ethics Committee of Beijing University of Technology. This study was a retrospective analysis of the “Study on Evaluation Method of Cardiovascular Disease Based on Non-invasive Detection of Blood Pressure and Pulse-Wave of Limbs” (Song et al., 2016), which recruited 412 subjects, where the clinic data collection as shown in Figure 1A. Herein, the Fukuda VS-1500A, manufactured by Beijing Fukuda Electronic Medical Instrument Co., Ltd, obtained the pulse waveform of four limbs, synchronously measures the blood pressure of four limbs, and automatically calculates the pulse wave velocity, ankle-brachial index, cardio-ankle vascular index, etc. CHM-T3002 Cardiac Hemodynamic Monitor, manufactured by Shandong Baolihao Medical Appliances Ltd., were used for measuring SV, AC, TPR, cardiac output, cardiac index, stroke volume index, ejection fraction, function index of left ventricular, index of contractility, etc. All subjects were registered at the Beijing University of Technology Hospital, and information on their diseases was collected from their medical records. The content of the study was explained to the subjects in detail, following which they signed an informed consent form based on this information. All experiments were performed per relevant guidelines and regulations.

### 2.2 Pulse waveform processing

The pulse waveform processing as shown in Figure 1B. Pulse waveform of four limbs was collected using a blood pressure pulse instrument. The pulse waveform obtained by the instrument was the curve of the blood vessel radius changing with time, and the waveform signal is typically expressed as a voltage value. Therefore, the voltage information was calibrated as the waveform information according to the following equation.

$$P\left(i\right)=\frac{P_{s}-P_{d}}{M_{s}-M_{d}}*\left[M\left(i\right)-M_{d}\right]+P_{d}\;\left(mmHg\right)$$ (1)

where $M_{s}$ , $M_{d}$ , and $M\left(i\right)$ are the peak value of the actual sampled signal, valley value of the actual sampled signal, and value of any point in the actual sampling signal, respectively; and $P_{s}$ , $P_{d}$ , and $P\left(i\right)$ are the systolic blood pressure, diastolic pressure, and any point pressure of the pulse wave, respectively.

The frequency domain information of the pulse wave was obtained using Fourier transform, which loses relatively less information. The physiological and pathological information contained in the waveform was retained as much as possible.

This study collected pulse signals from four limbs of the subjects, and approximately five–six complete waveforms were collected for each sample. To avoid subjectivity in the manual selection, the waveforms were averaged and normalized using a method reported previously (Li et al., 2019). To prevent the distortion of the pulse signals, according to the actual sampling frequency, the sampling points of one cardiac cycle of the pulse wave were set at 200. Because the focus of the model was on the characteristic information of the pulse wave, the amplitude of the pulse wave was normalized to 0–200 in each cycle.

### 2.3 Non-linear pulse wave theory model

The governing equation of blood flow was established based on continuum equations, Navier–Stokes equations and the basic assumptions of the NonPWT. The basic assumptions of NonPWT were as follows. Blood is a viscous and incompressible Newtonian fluid, and its flow in the artery is a symmetrical laminar flow ( $\frac{\partial^{2}u}{{\partial z}^{2}}\ll \frac{\partial^{2}u}{{\partial r}^{2}}$ ). The blood vessel wall is a thin-walled axisymmetric cone, that is, locally orthogonal, anisotropic, and elastically incompressible. The radial motion of the blood vessel wall is greater than that of the blood vessel wall, and the radial motion is $\frac{\partial R}{\partial t}$ . The axial and radial velocities vary with time at different locations in the blood vessel. The governing equation of blood flow is as follows.

$$\frac{\partial u}{\partial t}+u\frac{\partial u}{\partial z}+\vartheta \frac{\partial u}{\partial r}=-\frac{1}{\rho }\frac{\partial P}{\partial z}+\nu \left(\frac{\partial^{2}u}{{\partial z}^{2}}+\frac{\partial^{2}u}{{\partial r}^{2}}+\frac{1}{r}\frac{\partial u}{\partial r}\right)$$ (2)

$$\frac{\partial \vartheta }{\partial t}+u\frac{\partial \vartheta }{\partial z}+\vartheta \frac{\partial \vartheta }{\partial r}=-\frac{1}{\rho }\frac{\partial P}{\partial z}+\nu \left(\frac{\partial^{2}\vartheta }{{\partial z}^{2}}+\frac{\partial^{2}\vartheta }{{\partial r}^{2}}+\frac{1}{r}\frac{\partial \vartheta }{\partial r}-\frac{\vartheta }{r}\right)$$ (3)

$$\frac{\partial u}{\partial z}+\frac{1}{r}\frac{\partial }{\partial r}\left[rv\right]=0$$ (4)

where u and ϑ are the axial and radial velocities in the blood, respectively; ν is the blood viscosity; ρ is the blood density and $\rho =1.05*10^{-3}kg/cm^{3}$ ; and P is the blood pressure.

In this study, aortic blood flow was used as the basis for calculating HDIs. Therefore, the governing equation of blood flow requires further derivation until it can be solved. Based on the basic assumptions of NonPWT, radial coordinate normalization was introduced into the coordinate transformation, as follows.

$$\eta =\frac{r}{R\left(z,t\right)}$$ (5)

where $R\left(z,t\right)$ is the arterial radius; and η is the radial coordinate parameter. The boundary conditions were set as follows.

$$when\;r=R,\eta =1,u\left(1,z;t\right)=0,\vartheta \left(1,z;t\right)=\frac{\partial R}{\partial t}$$ (6)

$$when\;r=0,\eta =0,\frac{\partial u}{\partial r}\;{|}_{r=0}=\vartheta {|}_{r=0}=0$$ (7)

Based on these boundary conditions and assumptions, the basic equation for blood flow can be obtained as follows.

$$\frac{\partial u}{\partial t}=F\left(z,t\right)+\left(\frac{\eta }{R}\frac{\partial R}{\partial t}-\frac{\vartheta }{R}\right)\frac{\partial u}{\partial R}+\frac{u}{R}\left(\frac{\partial \vartheta }{\partial \eta }+\frac{\vartheta }{\eta }\right)+\frac{\nu }{R^{2}\eta }\frac{\partial }{\partial \eta }\left(\eta \frac{\partial u}{\partial \eta }\right)$$ (8)

where $F\left(z,t\right)$ is the axial pressure gradient; herein, $F\left(z,t\right)=-\frac{1}{\rho }\frac{\partial P}{\partial z}$

The basic equation of blood flow was calculated using a differential algorithm and numerical analysis, and the blood flow equation in the blood vessel in a cardiac cycle was solved as follows.

$$\frac{dQ}{dt}+\lambda Q+\varepsilon Q^{2}=AF\left(z,t\right)=-\frac{A}{\rho }\frac{\partial P}{\partial z}$$ (9)

which was the basis for calculating HDIs. Here, Q is the blood flow corresponding to each sampling point in the cardiac cycle; λ is the primary coefficient of blood flow change with time in a cardiac cycle, with $\lambda =\frac{8a\nu }{R^{2}}-\frac{4\beta }{R}\frac{\partial R}{\partial t}$ ; ε is the quadratic coefficient of blood flow change with time in a cardiac cycle, with $\varepsilon =-\frac{4\left(\beta +\beta_{0}\right)}{\pi R^{3}}\frac{\partial R}{\partial z}$ ; And A is a function of the change in the vessel radius with time during the contraction and expansion of the vessel wall in a cardiac cycle, with $A=\pi {R\left(t\right)}^{2}$ .

### 2.4 Establishment of pressure gradient model

This study solved the pressure gradient to calculate blood flow. The pressure gradient can be expressed as a linear superposition of harmonics with different amplitudes and frequencies, as follows.

$$P\left(z,t\right)=P_{m}+\overset{\infty }{\underset{n=1}{\sum }}A_{n}\exp\left[iw_{n}\left(t-\frac{z}{c_{n}}\right)\right]$$ (10)

where $P_{m}$ is the mean arterial blood pressure; $A_{n}$ is the amplitude of the harmonic; $w_{n}$ is the frequency of the harmonics, with $\omega_{n}=\frac{2n\pi }{T}$ ; And $c_{n}$ is the propagation velocity of the pressure wave.

In this study, the data sources were the blood pressure and pulse information of the four limbs. the corresponding pressure gradient calculation formula for the four limbs was obtained. The pressure gradient calculation formula at z=0 was derived by differentiating z and t in the formula:

$${{F\left(t\right)}_{f}=\left(\frac{\partial p}{\partial z}\right)}_{f}=-\frac{1}{c_{1f}}{\left(\frac{dP\left(t\right)}{dt}\right)}_{f}{\left[1+\sum_{n=1}^{\infty }\left(b_{nr}\cos\left(\omega_{n}t\right)-b_{nI}\sin\left(\omega_{n}t\right)\right)\right]}_{f}$$ (11)

where $c_{1}$ is the fundamental wave and propagation velocity of the pressure wave, with $c_{1}=\sqrt{\frac{R}{2\rho }\frac{\partial P}{\partial R}}$ ; $b_{nr}$ and $b_{nI}$ are the recurrence coefficients of the arterial pulse wave, with $b_{nr}=u_{n+1}\left(\frac{c_{1}}{c_{n+1}}-1\right)-\overset{n}{\underset{h=1}{\sum }}\left(u_{h}b_{\left(n-h\right)r}-v_{h}b_{\left(n-h\right)I}\right)$ and $b_{nI}=v_{n+1}\left(\frac{c_{1}}{c_{n+1}}-1\right)-\overset{n}{\underset{h=1}{\sum }}\left(v_{h}b_{\left(n-h\right)r}+u_{h}b_{\left(n-h\right)I}\right)$ ; and f is the label of the four limbs, with f=1,2,3, and 4 representing the right upper, left upper, right lower, and left lower limbs, respectively.

### 2.5 Calculation and derivation of blood flow

The blood flow equation in the cardiac cycle, which was introduced into the formal variable $\left(Q,t\right)=A∙F\left(t\right)-\lambda Q-\varepsilon Q^{2}$ , and the flow estimation and correction values were calculated using the estimation correction method.

$$Q_{n+1}^{*}=Q_{n}+TG\left(Q_{n},\tau_{n}\right)∆\tau$$ (12)

$$Q_{n+1}=Q_{n}+T\left[G\left(Q_{n},\tau_{n}\right)+G\left(Q_{n+1}^{*},\tau_{n+1}\right)\right]∆\tau /2$$ (13)

where $Q_{n+1}^{*}\;$ is the flow-estimation value; $Q_{n+1}$ is the flow correction value; n is the time sequence number, with n=1,2,……N ; τ is the time unit, with $\tau =\frac{t}{N},∆\tau =\frac{1}{N},\tau_{n}=\left(n-1\right)∆\tau$ ; and N is the number of sampling points in a cardiac cycle, which is equal to the number of sampling times in a cardiac cycle.

According to the blood flow equation and flow estimation and correction values, the blood flow equation of the four limbs in the cardiac cycle was derived and calculated as follows.

$${\left(\frac{dQ\left(t\right)}{dt}\right)}_{f}+{\lambda \left(t\right)}_{f}{Q\left(t\right)}_{f}+{\varepsilon \left(t\right)}_{f}{Q^{2}\left(t\right)}_{f}={A\left(t\right)}_{f}{F\left(t\right)}_{f}$$ (14)

The equation included three important parameters: ${\lambda \left(t\right)}_{f},{\varepsilon \left(t\right)}_{f},and\;{A\left(t\right)}_{f}$ . Herein, ${\lambda \left(t\right)}_{f}$ was solved as

$${\lambda \left(t\right)}_{f}=\frac{8\alpha \gamma }{{R^{2}\left(t\right)}_{f}}-\frac{\beta \left[\beta_{1}^{2}{\left(\alpha_{2}\beta_{2m}\right)}^{4}-1\right]}{P_{mf}-\left[\beta_{1}^{2}{\left(\alpha_{2}\beta_{2m}\right)}^{4}-1\right]\left({P\left(t\right)}_{f}-P_{mf}\right)}{\left(\frac{dP\left(t\right)}{dt}\right)}_{f}$$ (15)

where $P_{mf}$ is the mean arterial pressure of the four limbs in a cardiac cycle, with $P_{mf}=\frac{1}{T}\int_{0}^{T}{P\left(t\right)}_{f}dt$ ; ${P\left(t\right)}_{f}$ is the pulse pressure value of the four limbs at each sampling point, corresponding to each time in a cardiac cycle; α is the non-linear pulse-wave propagation coefficient of the flow rate with time; β is the non-linear pulse wave propagation coefficient with a time-varying blood-vessel radius; γ is the ratio of the blood dynamic viscosity to the blood density under the action of gravity; $\beta_{1}$ is the ratio of the blood vessel’s length *in vivo* and equilibrium; $\beta_{2m}$ is the ratio of the radius of the blood vessel to the undeformed radius of the blood vessel at equilibrium; and $\alpha_{2}$ is the correction coefficient under physiological human conditions.

Herein, ${\varepsilon \left(t\right)}_{f}$ was calculated as follows.

$${\varepsilon \left(t\right)}_{f}=\frac{4\left(\beta +\beta_{0}\right)}{\pi {R^{2}\left(t\right)}_{f}}\left(\frac{\tan\varphi }{{R\left(t\right)}_{f}}+\frac{\beta_{1}^{2}{\left(\alpha_{2}\beta_{2m}\right)}^{4}-1}{4{c\left(t\right)}_{f}\left\{P_{mf}-\left[{\beta_{1}^{2}\left(\alpha_{2}\beta_{2m}\right)}^{4}-1\right]\left({P\left(t\right)}_{f}-P_{mf}\right)\right\}}\;{\left(\frac{dP\left(t\right)}{dt}\right)}_{f}\right)$$ (16)

where φ is the half-cone angle of the blood vessel in its natural state; and $\beta_{0}$ is the non-linear pulse wave propagation coefficient with a time-varying blood-vessel radius.

Moreover, ${A\left(t\right)}_{f}$ was calculated as:

$${A\left(t\right)}_{f}=\pi {R^{2}\left(t\right)}_{f}=\pi {\left[R_{m}*\sqrt{1+b_{f}*\ln\frac{{P\left(t\right)}_{f}}{P_{mf}}}\right]}^{2}$$ (17)

where $R_{m}$ is the estimated human blood vessel radius, with $R_{m}=0.042+0.000625\left(1+0.36\sqrt{G}\right)\sqrt{hl}$ .

The blood flow of four limbs is related to the geometric shape of blood vessels. In order to accurately calculate the blood flow of four limbs, necessary animal experiments are carried out to estimate the correction coefficient, which are used to calculate the radius of four limbs.

### 2.6 Establishment of blood flow models based on blood pressure and pulse wave of four limbs

In this study, blood flow was key to the accuracy of the hemodynamic parameters. Due to systolic or diastolic blood pressure, blood pressure of upper or lower limbs shown different clinical applications when evaluating cardiovascular function. As reported in the study (Clark et al., 2012a; Chen et al., 2012; Cao et al., 2014), the differential pressure of upper limb systolic pressure is more than 15 mmHg, reminding patients to conduct further vascular assessment, which is a useful indicator of vascular disease and death risk. Based on the above theoretical model, as shown in the Figure 1C, we derive blood flow equation for different times in one cardiac cycle considering the different distributions of blood pressure and pulse wave of four limbs, then obtain the average blood flow in one cardiac cycle. The blood flow models established in four different conditions are defined as model I, model II, model III and model IV.

(1) Model I: Blood flow at different times in a cardiac cycle was calculated according to the blood flow of the four limbs at different times in a cardiac cycle.

$$Q\left(t\right)=a{Q\left(t\right)}_{1}+{bQ\left(t\right)}_{2}+{cQ\left(t\right)}_{3}+{dQ\left(t\right)}_{4}+e$$ (18)

where a,b,c, and d are the correction coefficients of the blood flow in the right upper, left upper, right lower, and left lower limbs, respectively; and e is a constant.

(2) Model II: Blood flow at different times in a cardiac cycle was calculated according to the high-order fitting of the sum of the four limbs.

$$Q\left(t\right)=a{\left.{\left(Q\left(t\right)\right.}_{1}+{Q\left(t\right)}_{2}+{Q\left(t\right)}_{3}+{Q\left(t\right)}_{4}\right)}^{2}+{b(Q\left(t\right)}_{1}+{Q\left(t\right)}_{2}+{Q\left(t\right)}_{3}+{Q\left(t\right)}_{4})+c$$ (19)

where a and b are the quadratic and first-order fitting coefficients, respectively; and c is a constant.

(3) Model III: Blood flow at different times in a cardiac cycle was calculated according to non-linear regression analysis of the higher systolic blood pressure in the upper and lower limbs.

$$Q\left(t\right)=a\left(\max\left({Q\left(t\right)}_{1},{Q\left(t\right)}_{2}\right)\right)+b\left(\max\left({Q\left(t\right)}_{3},{Q\left(t\right)}_{4}\right)\right)+c$$ (20)

where a and b are correction coefficients of the upper and lower limb blood flows, respectively; and c is a constant.

(4) Model IV: Blood flow at different times in a cardiac cycle was calculated according to the high-order fitting of the sum of the higher systolic blood pressure in the upper and lower limbs.

$$Q\left(t\right)=a{\left(\max\left({Q\left(t\right)}_{1},{Q\left(t\right)}_{2}\right)+\max\left({Q\left(t\right)}_{3},{Q\left(t\right)}_{4}\right)\right)}^{2}$$

$$+b\left(\max\left({Q\left(t\right)}_{1},{Q\left(t\right)}_{2}\right)+\max\left({Q\left(t\right)}_{3},{Q\left(t\right)}_{4}\right)\right)+c$$ (21)

where a and b are the quadratic and first-order fitting coefficients, respectively; and c is a constant.

### 2.7 Model establishment of cardiovascular hemodynamic indexes

Cardiovascular hemodynamic parameters can comprehensively evaluate the health of the CVS. Due to the early clinical symptoms of CVDs are not obvious, the probability of undetected high-risk patients is still high. The study used three hemodynamic diagnosis indexes that can reflect cardiovascular function easily and quickly. SV directly represented the outcome of heart pumping ability and was a perioperative monitoring indicator during cardiovascular surgery. AC reflects the ability of the artery to passively expand during ventricular systole to accommodate most of the stroke volume while continuing blood flow during diastole. TPR is an important hemodynamic indicator that reflects cardiac afterload. These are the main hydrodynamic factors that determine high and low arterial blood pressures. Herein, SV,TPR,and AC were solved using the following equations.

$$SV=\int_{0}^{T}Q\left(t\right)dt=Q_{m}T,\left(ml/B\right)$$ (22)

$$TPR=\frac{P_{m}}{CO}\left(\frac{mmHg}{ml}\right)=80\frac{P_{m}}{CO},\left(dyn∙s/cm^{5}\right)$$ (23)

$$AC=\frac{SV}{Ps-Pd},\left(ml/B/mmHg\right)$$ (24)

### 2.8 Data screening

**FIGURE 2:**
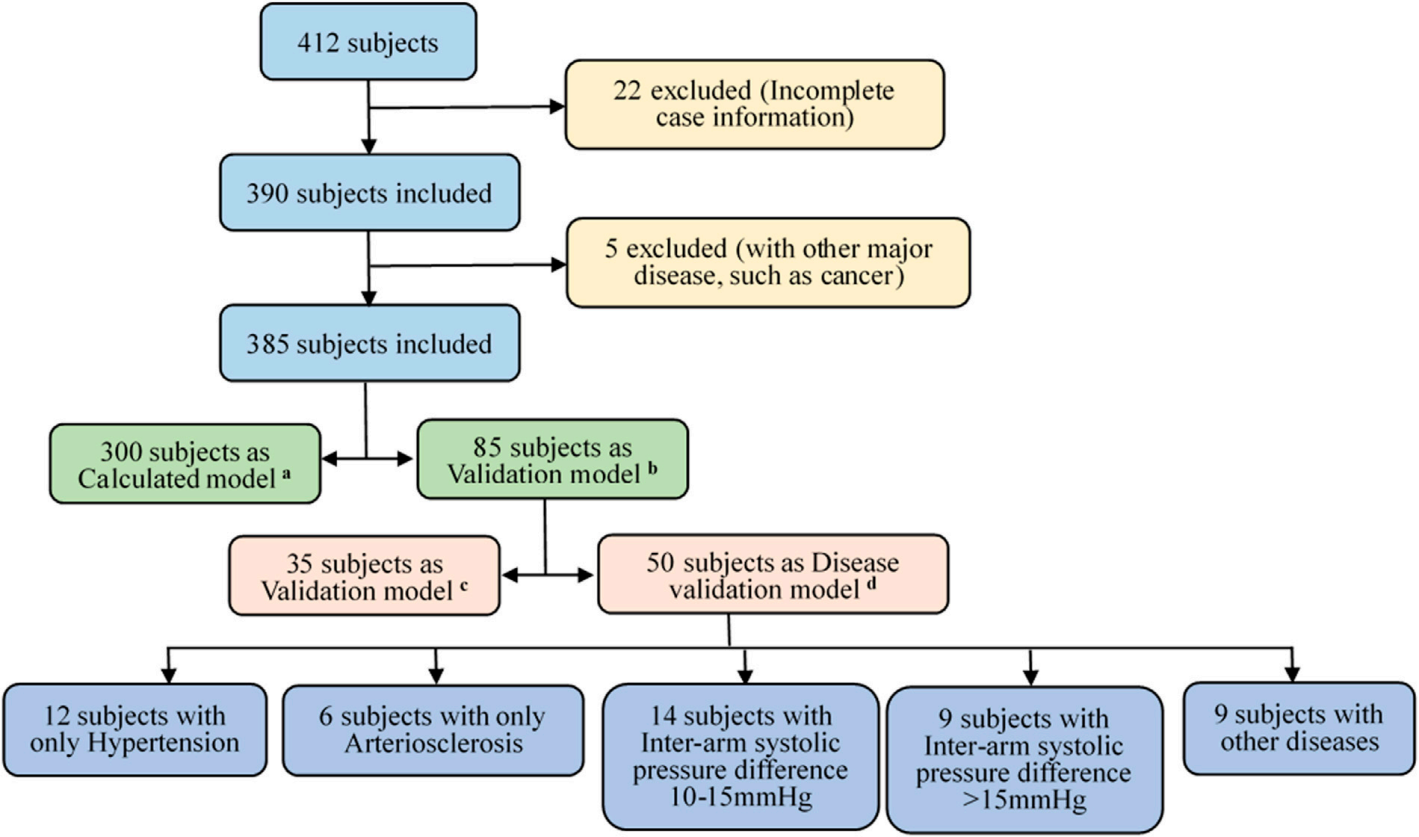
Subjects’ data screening process. Screening criteria **(A–D)**: **(A)** calculated model group of 300 subjects, **(B)** validation model group of 85 subjects; **(C)** verifying the accuracy of the model using 35 randomly-selected subjects, and **(D)** another 50 randomly-selected subjects and under different disease conditions.

Next, the proposed hemodynamic diagnosis method based on NonPWT was verified for universally applicability to people. The datasets were classified into three groups: A calculated and two validation model groups. Figure 2 illustrates the data-screening procedure. For the validation group, 35 subjects were randomly selected to verify the model accuracy. To further evaluate the capability of the HDIs proposed model, 50 subjects were selected to verify the model accuracy under different disease conditions. These 50 subjects fulfilled the following three criteria. 1) The detection of pressure and the pulse wave of four limbs and the cardiac function parameters were completed. 2) Patients with cancer, heart failure, and other uncommon CVDs that had significant effects on pulse waves and cardiac function parameters were excluded. 3) Inter-arm blood pressure differences (10–15 mmHg and >15 mmHg) were considered risk factors.

The experimental design and investigation of clinical data were based on epidemiology and the clinical data was statistically analyzed. The clinical HDIs results were compared with those of the proposed NonPWT model. Thus, the consistency between the algorithm in this study and clinical measurements was verified. Additionally, complex equations were calculated and solved.

## 3 Results

### 3.1 Calculation results of blood flow

**FIGURE 3:**
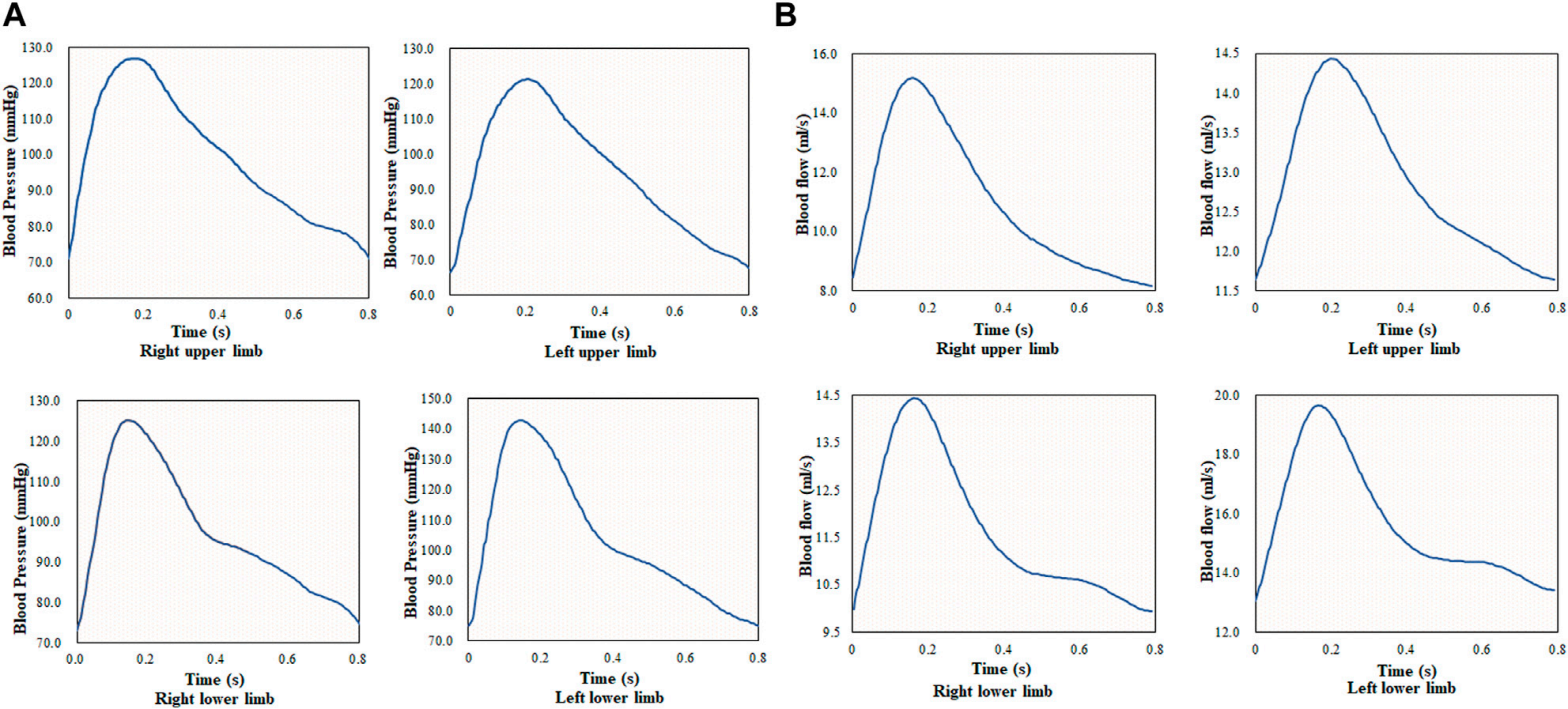
Pulse waveform and blood flow of four limbs during one cardiac cycle; **(A)** pulse waveform of four limbs during one cardiac cycle, and **(B)** blood flow of four limbs during one cardiac cycle.

Blood flow in the upper and lower arteries was calculated based on the pulse waveform of the four limbs in a cardiac cycle in Figure 3A, and the results are shown in Figure 3B. The pulse wave is a wave generated by a heartbeat and pumping of blood to the extremities and the terminal arteries. It is the waveform of the pressure generated by the blood flow against the walls of the blood vessels, and the waveform reflects the changes in the volume of circulating blood, with the peak of the wave reflecting the moment of maximum blood flow. In this study, data at the end of the extremities were collected under the guidance of the clinician, and the reference values for blood flow at the end of the upper and lower extremities in the clinic were 2.5–12.67 ml/s and 5.33–19.87 ml/s, respectively. The results revealed that the average blood flow in the cardiac cycle of the upper artery was 10.78 ml/s, and the blood flow in the lower artery was higher than that in the upper artery. Compared with the clinically measured values, the calculated value of blood flow was within the clinical reference range.

The blood flow of the four limbs and the blood flow of the body were analyzed with medical statistics using 300 cases; thus, the fitting formulas (models I, II, III, and IV) were obtained. The quadratic fitting formula of the sum of blood flow of the four limbs was *p* < 0.1. The quadric fitting formula of the sum of the higher systolic blood pressure in the upper and lower limbs was *p* < 0.05.

$$Q\left(t\right)=7.147{Q\left(t\right)}_{1}+{3.481Q\left(t\right)}_{2}+{0.347Q\left(t\right)}_{3}+{2.161Q\left(t\right)}_{4}+82.082$$ (25)

$$Q\left(t\right)=0.09{\left(Q\left(t\right)\right.}_{1}+{Q\left(t\right)}_{2}+{\left.{Q\left(t\right)}_{3}+{Q\left(t\right)}_{4}\right)}^{2}-8.522{\left(Q\left(t\right)\right.}_{1}+{Q\left(t\right)}_{2}+{Q\left(t\right)}_{3}+{Q\left(t\right)}_{4})+302.177$$ (26)

$$Q\left(t\right)=2.547\left(\max\left({Q\left(t\right)}_{1},{Q\left(t\right)}_{2}\right)\right)+0.32\left(\max\left({Q\left(t\right)}_{3},{Q\left(t\right)}_{4}\right)\right)+77.029$$ (27)

$$Q\left(t\right)=0.393{\left(\max\left({Q\left(t\right)}_{1},{Q\left(t\right)}_{2}\right)+\max\left({Q\left(t\right)}_{3},{Q\left(t\right)}_{4}\right)\right)}^{2}-18.817(\max\left({Q\left(t\right)}_{1},{Q\left(t\right)}_{2}\right)$$

$$+\max\left({Q\left(t\right)}_{3},{Q\left(t\right)}_{4}\right))+324.143$$ (28)

### 3.2 Calculation results of hemodynamic diagnosis indexes

Herein, 35 cases were randomly selected from the sample, and a t-test was performed. The calculation results for SV, TPR, and AC are summarized in Table 1. Compared with the clinical measurement value, SV and TPR exhibited no significant difference between the four calculated models, whereas for AC, only model IV was not significantly different. The SV was the least significant difference in the statistical results for model IV; Hence, the calculated results of SV were closest to the clinical results, followed by TPR and AC. Based on the results of the fit of the computational model, the quadratic fitting formula of the sum of the higher systolic blood pressure in the upper and lower limbs was statistically significant, and the fitted model was closer to the measured values than the simple linear model. After the combined evaluation, model IV was used as a model for cardiovascular disease with different risk factors.

**TABLE 1:** Comparison calculation and clinical measurements value of SV, TPR, and AC.

Datasets	SV(ml)	p	TPR(dyn∙s/cm5)	p	AC(ml/mmHg)	p
Model I	73.73 ± 15.90	0.955	1506.55 ± 138.01	0.683	2.02 ± 0.27	0.009
Model II	72.83 ± 14.28	0.941	1467.28 ± 159.19	0.864	2.12 ± 0.32	0.048
Model III	72.33 ± 15.18	0.885	1503.65 ± 175.75	0.746	2.03 ± 0.26	0.009
Model IV	73.63 ± 15.81	0.966	1470.35 ± 181.29	0.906	2.12 ± 0.33	0.051
Clinical value	73.33 ± 15.27		1479.40 ± 153.83		2.45 ± 0.37	

Data are presented as mean ± SD, and *p*-values are calculated based on the independent sample t-test. SV, stroke volume; TPR, total peripheral resistance; AC, arterial compliance.

**FIGURE 4:**
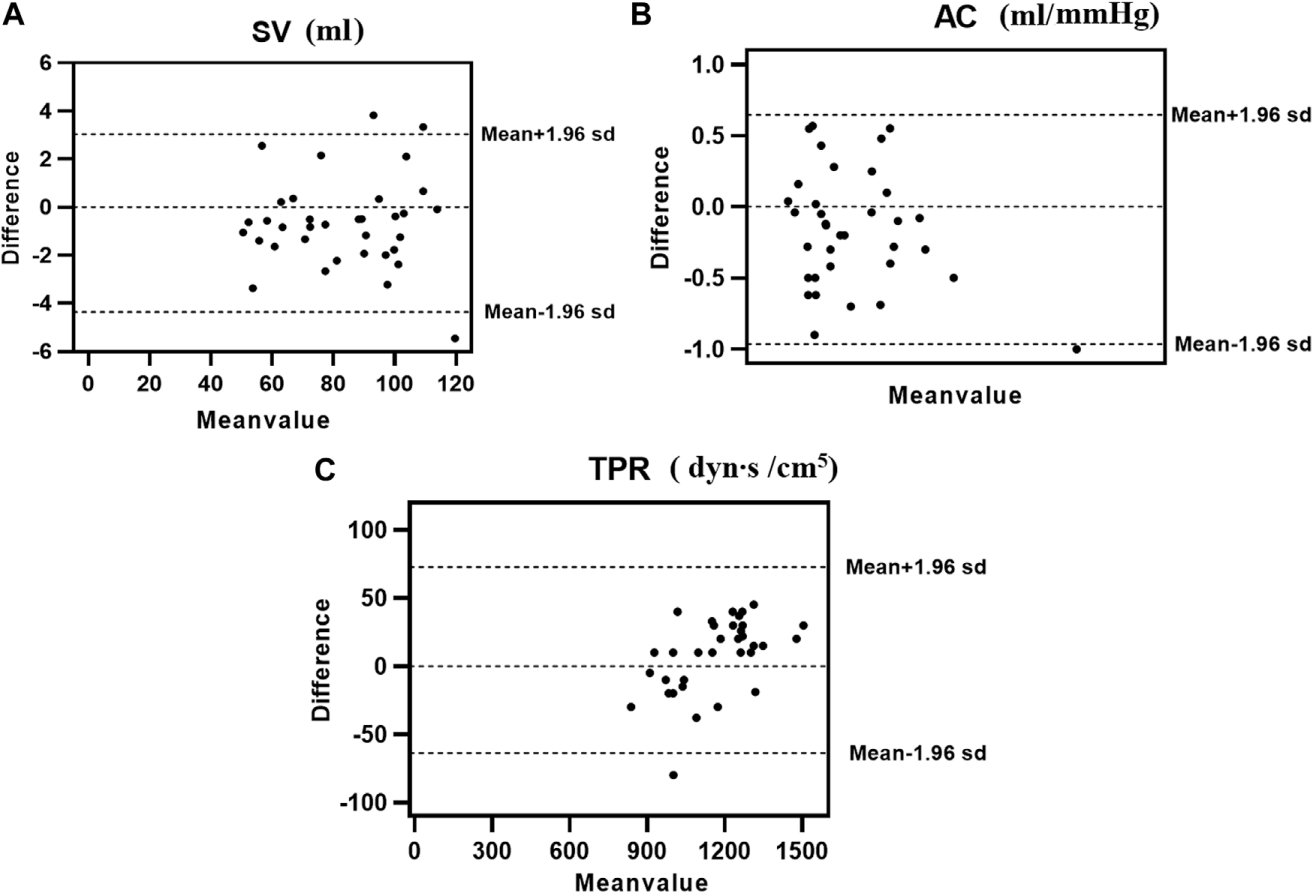
Consistency analysis between calculation and clinical measurements value of **(A)** SV, **(B)** AC, and **(C)** TPR; The relationship between the average (horizontal axis) and the difference (vertical axis) is illustrated using scatter plots.

The consistency test between the calculated and clinical measurement results was further verified through the Bland–Altman method-based analysis based on the model IV values of SV, TPR, and AC. The results are shown in Figure 4; The relationship between the average (horizontal axis) and difference (vertical axis) is illustrated using scatter plot. According to the basic idea of the Bland–Altman method, the two methods were generally consistent when 95% more points existed in the scatter plot within the confidence interval (mean + 1.96 standard deviation; Mean—1.96 standard deviation) that did not exceed the professionally acceptable critical value range. For SV, TPR, and AC, 35 sets fell within the 95% confidence interval, and the results of the two methods were consistent. Thus, these results indicated that the calculated model based on the NonPWT was consistent with the clinical measurements. Note that for clinical applications, for a clinical requirement that the difference between the two methods must fall within a certain range, the bounded range of consistency will be reconsidered, and consistency will be re-evaluated, which is expected to be further improved using larger datasets as our future task.

### 3.3 Calculation results of hemodynamic diagnosis indexes with cardiovascular diseases

A risk factor for a disease is a factor that increases the incidence of the disease in a population, and when this factor is removed, the incidence of the disease decreases. Hypertension, atherosclerosis, and inter-arm systolic blood pressure differences are major risk factors for cardiovascular disease. To verify the accuracy and applicability of the calculated model, the subjects were divided into four groups and the results with the calculations of model IV were compared. Fifty cases were randomly selected from the sample; they were divided into four groups according to Figure 2; Subsequently, a t-test was performed. As summarized in Table 2, the mean values of the five groups in terms of systolic and diastolic blood pressures were significantly different (*p* < 0.001). The risk factors for each disease group significantly differed from those of the control group. However, no significant differences were observed in HDIs (SV, TPR, and AC) between any of the disease and control groups after performing the t-test.

**TABLE 2:** Basic characteristics in cardiovascular diseases.

Characteristics	300 subjects	Atherosclerosis	Hypertension	IASBPD	IASBPD
				10–15 mmHg	>15 mmHg
Age	60.56 ± 8.69	61.83 ± 4.88	61.75 ± 4.58	59.86 ± 4.40	57.22 ± 12.51
BMI(kg/m^2^)	25.43 ± 3.60	22.43 ± 2.78*	28.05 ± 3.78**	25.55 ± 3.09	25.16 ± 3.28
SBP(mmHg)	136.08 ± 18.52	129.83 ± 7.33*	162.00 ± 19.58**	124.86 ± 6.25*	122.00 ± 8.69*
DBP(mmHg)	81.83 ± 10.59	77 ± 6.03	95.75 ± 9.02**	77.07 ± 7.89*	71.67 ± 10.39**
CAVI	7.76 ± 1.21	7.45 ± 0.93	7.90 ± 1.52	7.42 ± 0.93	8.04 ± 0.73**
ABI	1.08 ± 0.11	0.77 ± 0.20**	1.01 ± 0.07**	1.01 ± 0.19	0.94 ± 0.19
SV(ml)	77.38 ± 18.12	88.38 ± 20.25	68.54 ± 12.97*	73.87 ± 14.47	87.95 ± 19.33
TPR(dyn∙s/cm^5^)	1569.79 ± 420.73	1299.75 ± 496.20	1739.79 ± 354.68	1557.92 ± 399.32	1317.49 ± 348.46
AC(ml/mmHg)	1.73 ± 0.77	2.03 ± 0.51	1.49 ± 0.62	1.73 ± 0.60	2.09 ± 0.54

Data are presented as mean ± SD; *p* values: Significant differences are calculated based on the independent samples t-test; **p values < 0.001; *p values < 0.05. IASBPD, inter-arm systolic blood pressure difference; BMI, body mass index; CAVI, cardio-ankle vascular index; ABI, ankle-brachial index; SBP, systolic blood pressure; DBP, diastolic blood pressure; SV, stroke volume; TPR, total peripheral resistance; AC, arterial compliance.

After the classification according to risk factors, t-tests were performed on clinical measurements and calculated values. Model IV was used as the computational model because it was closest to the clinical values among the four models. The mean SV, TPR, and AC values are summarized in Table 3. Although no significant differences were observed between the statistical results and the clinical measurement values, a new phenomenon was observed, as follows. The *p* values of SV in the inter-arm systolic pressure difference >20 mmHg and in the hypertensive group were smaller, thus impacting the accuracy of the SV calculation when the subject had a hypertensive disease or an inter-arm systolic pressure difference >20 mmHg. The *p*-value of TPR was smallest for inter-arm systolic pressure differences >20 mmHg; therefore, when the inter-arm systolic pressure difference of the subject was greater than 20 mmHg, a greater impact was reflected on the accuracy of the TPR calculation. The *p*-value of AC for inter-arm systolic pressure difference >20 mmHg was the smallest; therefore, when the inter-arm systolic pressure difference of the subject was greater than 20 mmHg, a greater impact was reflected on the accuracy of the AC calculation. Both TPR and AC reflect the vascular pliability function, which is corroborated by previous studies that suggest a risk of atherosclerosis when the inter-arm systolic pressure difference is greater than 20 mmHg.

**TABLE 3:** Comparison calculation and clinical measurements value of SV, TPR, and AC with cardiovascular diseases.

	Datasets	SV(ml)	p	TPR(dyn∙s/cm^5^)	p	AC(ml/mmHg)	p
Atherosclerosis	Model IV	85.27 ± 19.43	0.792	1488.83 ± 514.86	0.532	1.95 ± 0.53	0.792
	Clinical value	88.38 ± 20.25		1299.75 ± 496.20		2.03 ± 0.51	
Hypertension	Model IV	67.03 ± 10.94	0.761	1828.25 ± 445.13	0.595	1.41 ± 0.49	0.731
	Clinical value	68.54 ± 12.97		1739.79 ± 354.68		1.49 ± 0.62	
IASBPD 10–15 mmHg	Model IV	72.46 ± 14.76	0.800	1663.41 ± 452.32	0.519	1.63 ± 0.59	0.65
	Clinical value	73.87 ± 14.47		1557.92 ± 399.32		1.73 ± 0.60	
IASBPD >15 mmHg	Model IV	84.27 ± 17.99	0.682	1468.60 ± 390.68	0.399	1.95 ± 0.41	0.537
	Clinical value	87.95 ± 19.33		1317.49 ± 348.46		2.09 ± 0.54	

Data are presented as mean ± SD, and p values are calculated based on the independent sample t-test. SV, stroke volume; TPR, total peripheral resistance; AC, arterial compliance.

**FIGURE 5:**
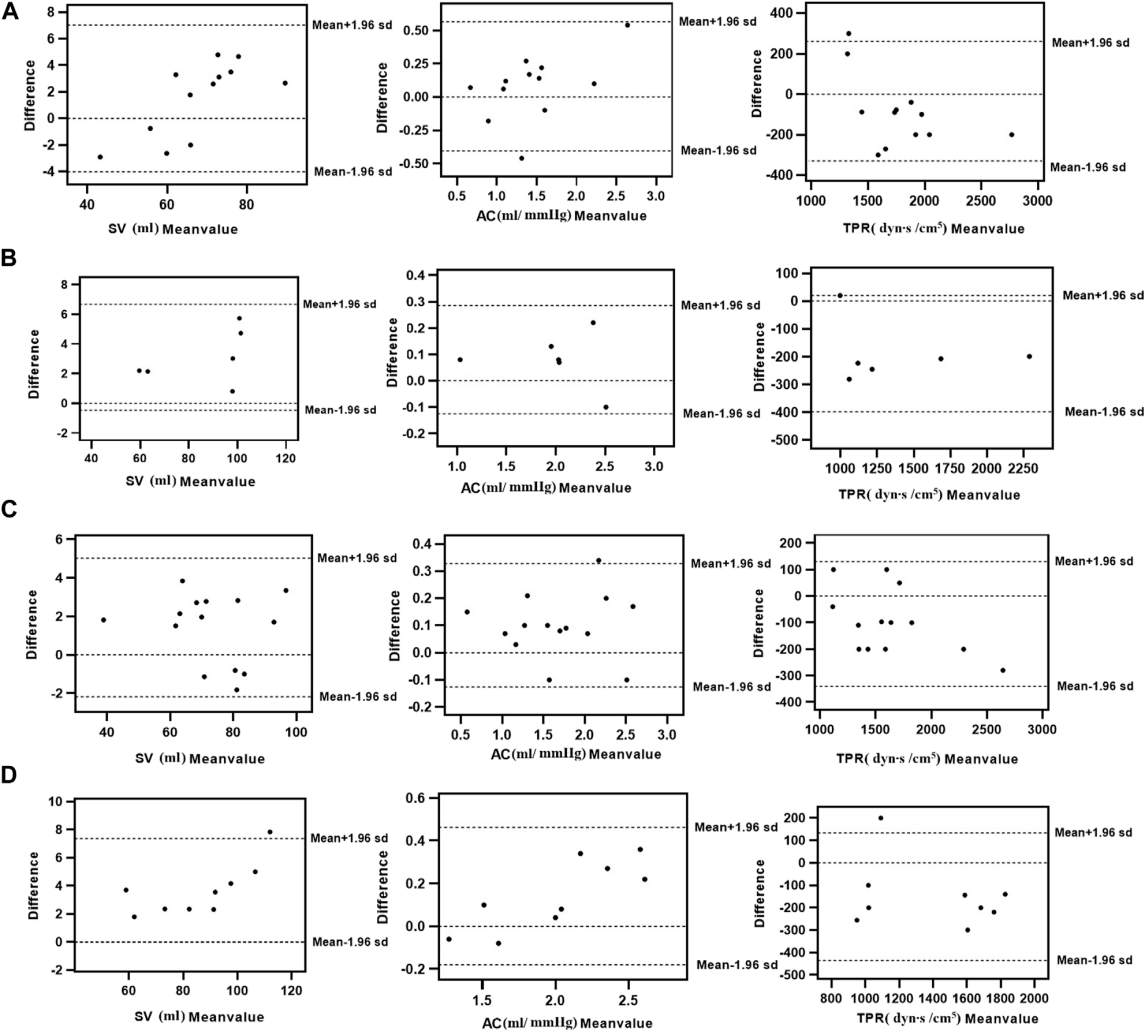
Consistency analysis between calculation and clinical measurements value of SV, TPR, and AC with cardiovascular diseases [**(A)** Hypertension, **(B)** Atherosclerosis, **(C)** IASBPD 10–15 mmHg, and **(D)** IASBPD >15 mmHg]; The relationship between the average (horizontal axis) and the difference (vertical axis) is illustrated using scatter plots.

The consistency test between the calculated and clinical measurement results was further verified through the Bland–Altman method-based analysis, and the results are shown in Figure 5. According to the basic idea of the Bland–Altman method, the SV, TPR, and AC of these sets fall within the 95% confidence interval, and extremely rare cases will fall outside of the 95% confidence interval. Thus, our results demonstrated that the calculated model based on the NonPWT was consistent with clinical measurements in subjects with CVDs. However, the validation of disease factors revealed that risk factors led to a reduction in significant differences, which was expected to be further improved by disease classification in our future work.

## 4 Discussions

This study proposed a non-invasive algorithm to calculate HDIs based on the blood pressure and pulse wave of four limbs *via* the NonPWT. Compared with the previous pulse wave theory, the proposed NonPWT retained the non-linear characteristics of physiological information and frequency domain characteristics of the waveform, and realized fast and accurate calculation of HDIs. Compared with the relative error, the calculation error of the sum of the higher systolic blood pressures in the upper and lower limbs was the smallest. Consistency analysis revealed that 95% of the points of the HDIs fell within the limits of agreement. Additionally, it exhibited practical value in the HDIs calculation, which could considerably improve the computational efficiency and simplify the operation process.

The computational models of HDIs are based predominantly on the pulse wave information of a single limb; however, the human body is a complex physiological system, and blood pressure formation and fluctuations are achieved through the dynamic interaction between the heart and vascular system. At the functional level, numerous factors, such as blood flow, output per beat, vessel wall elasticity, and peripheral resistance, influence blood pressure. Studies (Charmoy et al., 2007; Verberk et al., 2011; van der Hoeven et al., 2013) have shown that blood pressure measurement affects the diagnosis of CVDs. Moreover, some studies (O’Shea and Murphy, 2000; Clark et al., 2012b; Mancia et al., 2013; Singh et al., 2015) have also confirmed that simultaneous measurement of blood pressure in all four limbs and evaluation of blood pressure differences in all four limbs can provide additional clinical information that can contribute to clinical decision-making and predict clinical prognosis as a major indicator. In particular, the difference in systolic blood pressure in both upper extremities and the difference in systolic blood pressure in both lower extremities of >15 mmHg can cause peripheral vascular disease and is typically associated with underlying CVDs (Mancia et al., 2013; van der Hoeven et al., 2013; Weber et al., 2014). In this study, we derive blood flow equation for different times of the cardiac cycle considering the four different distributions of blood pressure and pulse wave of four limbs. The higher second fit of the sum of the higher systolic blood pressure in the upper extremities and the higher systolic blood pressure in the lower extremities was statistically significant, and the calculation error of HDIs, as summarized in Table 1, revealed model IV to be the closest to the clinical values among the four models. In a comprehensive analysis, the calculation of HDIs using the information of blood pressure pulses of the four limbs was closer to the realism of cardiovascular health. Therefore, further studies on cardiovascular health assessment, both in mathematical models and hydrodynamic simulations, should consider the information on blood pressure and pulse wave of the four limbs in estimating blood flow.

Blood flow is key to calculating HDIs, and it directly affects the calculation results. This study proposed four models for blood flow considering the four different distributions of blood pressure and pulse wave of four limbs. Blood pressure and pulse wave data of the four limbs were used in models I and II, and blood pressure and pulse wave data of the four limbs that had higher systolic blood pressure among the four limbs were used in models III and IV. The statistical analysis results revealed that the quadratic fit formula of model IV was statistically significant (*p* < 0.05). The calculation error of HDIs, model IV, had the smallest error. According to Poiseuille’s law, blood flow is determined by a combination of factors, such as the radius, length, and pressure of blood vessels. The blood pressure pulse data of the lower extremity will be less accurate in calculating blood flow with changes in vessel length and radius, which explains why the calculation error of the HDIs of models I and II was larger. In addition, it also shows that the model has higher accuracy in calculating HDIs, which are closer to the heart. Because the blood vessels were elastic and their radius was variable, relying on the blood flow at a given moment to calculate the vascular compliance and total peripheral resistance led to the largest computational error with the radius of the vessel changes. Despite the error in the model, consistency was confirmed between the model based on NonPWT and actual clinical measurements, which illustrated the feasibility of the model in this study. Based on the blood pressure pulse information of the four limbs, the health detection of cardiovascular function was achieved, and its non-invasive detection method, simplicity, and affordability appeared to have more practical applications. This prevented people from going to the hospital only when they had symptoms of discomfort, which delayed the condition and missed the optimal treatment time.

Non-etheless, this study has several limitations. First, a major limitation lies in the small number of subjects and the related clinical information. The fitting coefficients for the calculation of blood flow were derived primarily from medical statistics. From a statistical perspective, the larger the sample size, the higher the accuracy of the fitting coefficients; the proposed blood flow model can create relatively high-quality calculation and accomplish a high-accurate HDIs calculation. In addition, taking into consideration that some physiological information, such as age, height, weight, and shoulder width, were involved in the blood flow model, which inevitably led to calculation errors due to the individual differences of subjects. In future work, we will classify subjects and build personalized models. Second, it was impossible to clarify whether the model is capable to specific functions which can reflect the systolic and diastolic functions of the heart. A diagnostically important application of the current method may be the calculation of SV, AC and TPR while quantitatively evaluating the severity of specific CVDs, which will be explored in our future work when large-size datasets are available and more cardiac function parameters are increased to be predictable. Third, this study focused on calculating the cardiac function parameters. Four blood flow models were constructed for this purpose. However, according to Poiseuille’s law, the brachial artery of the upper limb was closer to the heart than the ankle artery, and the resistance was smaller, which led to lower blood pressure in the brachial artery than in the ankle artery, thus leading to different blood pressures in the limbs. Although the larger the sample size, the higher the accuracy of statistics, it was hard to estimate how many additional samples are enough to improve the accuracy for the HDIs. Therefore, we should consider the weight of the blood flow of the four limbs and the classification of cardiovascular diseases to propose NonPWT methods to achieve more complex HDIs calculations in future work.

## 5 Conclusion

In this study, a model of pressure gradient and blood flow was built *via* NonPWT and a hemodynamic diagnosis model was proposed. On this basis, a fast and accurate calculation of HDIs was achieved. Compared with the traditional calculation method, the proposed NonPWT method retained the non-linear characteristics of the physiological information and frequency domain characteristics of the waveform and increased the computational accuracy. Compared with previous cardiovascular function research, the proposed cardiovascular hemodynamic diagnosis method extracted pulse wave data from four limbs and considered the risk factors of cardiovascular diseases, which implies higher accuracy. Thus, it can be applied for calculating other HDIs, in addition to those mentioned in the article, as well as those in other research fields assessing cardiovascular health. In terms of waveform data processing, computational efficiency, and accuracy, the proposed hemodynamic diagnosis method can meet the need for non-invasive calculations for cardiac health. It can be applied to the general health examination of healthcare institutions such as community medical care, rehabilitation and health care, sports and fitness, leisure and recuperation.

## Data Availability

The raw data supporting the conclusion of this article will be made available by the authors, without undue reservation.
